# Treatment Approaches to Chronic Lymphocytic Leukemia With High-Risk Molecular Features

**DOI:** 10.3389/fonc.2021.780085

**Published:** 2021-12-09

**Authors:** Lina van der Straten, Paul J. Hengeveld, Arnon P. Kater, Anton W. Langerak, Mark-David Levin

**Affiliations:** ^1^ Department of Internal Medicine, Albert Schweitzer Hospital, Dordrecht, Netherlands; ^2^ Laboratory Medical Immunology, Department of Immunology, Erasmus MC, Rotterdam, Netherlands; ^3^ Department of Research and Development, Netherlands Comprehensive Cancer Organisation (IKNL), Utrecht, Netherlands; ^4^ Department of Hematology, Amsterdam University Medical Center, University of Amsterdam, Cancer Center Amsterdam, Lymphoma and Myeloma Center Amsterdam, Amsterdam, Netherlands

**Keywords:** chronic lymphocytic leukemia, high-risk, treatment, *TP53*, del(11q), complex karyotype, unmutated IGHV, *NOTCH1*

## Abstract

The clinical course of chronic lymphocytic leukemia (CLL) is highly variable. Over the past decades, several cytogenetic, immunogenetic and molecular features have emerged that identify patients suffering from CLL with high-risk molecular features. These biomarkers can clearly aid prognostication, but may also be capable of predicting the efficacy of various treatment strategies in subgroups of patients. In this narrative review, we discuss treatment approaches to CLL with high-risk molecular features. Specifically, we review and provide a comprehensive overview of clinical trials evaluating the efficacy of chemotherapy, chemoimmunotherapy and novel agent-based treatments in CLL patients with *TP53* aberrations, deletion of the long arm of chromosome 11, complex karyotype, unmutated IGHV, B cell receptor stereotypy, and mutations in *NOTCH1* or *BIRC3*. Furthermore, we discuss future pharmaceutical and immunotherapeutic perspectives for CLL with high-risk molecular features, focusing on agents currently under investigation in clinical trials.

## Introduction

Chronic lymphocytic leukemia (CLL) is the most prevalent form of leukemia in the Western world with an age-standardized incidence rate of 4-5 cases per 100,000 persons per year (1–5). The clinical course of CLL is characterized by marked heterogeneity, with some patients surviving for more than 10 years without treatment, whereas others suffer rapid disease progression and poor outcome, in spite of the availability of effective treatment regimens. Historically, prognostication in CLL has relied on clinical staging: the Rai and Binet staging systems, developed approximately 40 years ago, are still frequently used in clinical practice (6, 7). However, as advanced molecular techniques such as fluorescence *in situ* hybridization (FISH), next-generation sequencing (NGS) and microarray-based genomic profiling have provided greater insight into the biology of CLL cells, the prognostication paradigm has shifted towards a perspective that not only relies on clinical features, but also incorporates high-risk genetic and molecular biomarkers. Indeed, the CLL international prognostic index (CLL-IPI) incorporates cytogenetic, immunogenetic and molecular features to predict the survival of CLL patients (8). In addition to prognostication, the presence of certain cytogenetic, immunogenetic and molecular features can predict differential responses to treatment. Several of the more newly discovered biomarkers are not routinely measured in clinical research and patient care, despite evidence of their predictive impact. Conversely, some routinely measured biomarkers that were historically associated with poor prognosis in the chemo(immuno)therapy era may have lost their predictive value in the context of novel agent-based therapies. Presently, the international workshop on CLL (iwCLL) guidelines recommend assessment of immunoglobulin heavy chain gene (IGHV) mutational status and *TP53* aberrations in every CLL patient before the initiation of treatment (9). *TP53* aberrations should be assessed by both targeted FISH and either Sanger sequencing or NGS. Whereas the IGHV mutational status is stable over time, additional (cyto)genetic abnormalities with therapeutic implications may be acquired over the course of the disease, necessitating reassessment at every subsequent line of therapy (9).

This narrative review discusses such therapeutic implications of CLL with high-risk molecular features. Specifically, we review the predictive impact, if any, of *TP53* aberrations, deletion of the long arm of chromosome 11, complex karyotype or genomic complexity, unmutated IGHV, B cell receptor stereotypy, mutated *NOTCH1* and mutated *BIRC3*. Furthermore, we discuss future perspectives for CLL with high-risk molecular features, focusing on upcoming agents in the therapeutic armamentarium of CLL.

## *TP53* Aberrations

Among the numerous prognostic and predictive biomarkers that have been identified over the previous decades, *TP53* aberrations indisputably remain the single most impactful genetic lesion in CLL. The *TP53* locus encodes the tumor-suppressor protein p53, which plays a key role in cell division, apoptosis and genomic stability. *TP53* signaling can be impaired through deletion of the *TP53* gene in the chromosomal locus 17p13.1, i.e. del(17p), or through genetic lesions, including missense and nonsense mutations, deletions, insertions or splice-site mutations. Cumulatively, these aberrations are present in 4-8% of all CLL patients at diagnosis, 10% at start of first-line therapy and 30-40% in relapsed or refractory (R/R) CLL patients who were previously treated with chemoimmunotherapy (10). In CLL patients, impaired *TP53* signaling is associated with a poor response to chemotherapy and chemoimmunotherapy. Chemotherapeutic agents exert their cytotoxic effects by causing DNA damage. In *TP53* wildtype cells, such irreparable damage results in apoptotic cell death. However, if *TP53* is not expressed or not functional, chemotherapy-induced DNA damage does not lead to apoptosis and as consequence, administration of these drugs leads to accumulation of detrimental genomic mutations while failing to induce cell death, possibly worsening disease prognosis.

### First-Line Setting

Indeed, after first-line treatment with fludarabine monotherapy (F) or fludarabine and cyclophosphamide (FC) in the CLL4 trial, conducted by the German CLL study group (GCLLSG), the overall survival (OS) of CLL patients was markedly shorter in patients harboring *TP53* mutations, compared with those without (median OS, 23.3 *versus* 62.2 months) (11, 12). The comparable E2997 (US) and LRF CLL4 (UK) trials yielded similar results (13, 14). In the phase III GCLLSG CLL8 trial, first-line treatment with FC was compared with fludarabine, cyclophosphamide and rituximab (FCR) (15, 16). While patients with del(17p) had relatively short PFS in both arms, median PFS in the FCR-arm was superior (FCR, 11.2 months *versus* FC, 9.1 months; hazard ratio [HR], 0.49; 95% CI, 0.25-0.93; *P*=0.02). Although FCR was the first treatment regimen that prolonged OS in CLL patients, the OS of patients with del(17p) was not significantly different between both arms (HR 0.66; 95%CI 0.33-1.31), underscoring the poor response in the *TP53* aberrant group. Indeed, multivariable analysis identified *TP53* aberrations as the strongest predictor of inferior PFS (HR 2.92; 1.78-4.78) and OS (HR 2.72, 1.60-4.60). Similarly, in a large Italian retrospective cohort study, patients with del(17p) had worse prognosis after FCR (median PFS, 22.5 months, compared with 58.9 months for patients without del(17p), HR 3.72; 95%CI, 2.42-5.72) and a high probability (19%) of developing secondary malignancies (17).

Based on the evidence from the trials discussed above, chemoimmunotherapy is no longer considered an acceptable first-line treatment for patients with *TP53* aberrations. Initially, the anti-CD52 monoclonal antibody (mAb) alemtuzumab, with or without methylprednisolone, was considered the only effective pharmaceutical treatment in patients with *TP53* aberrations, despite limited efficacy (overall response rate [ORR], 82%, median PFS 11.8 months, median OS 23 months) and an unfavorable toxicity profile (18). In addition, an allogeneic hematopoietic stem cell transplantation (alloHSCT) was considered as a first-line consolidation treatment for patients with *TP53* aberrations (19–21). The GCLLSG CLL3X trial demonstrated that the 6-year OS rate after alloHSCT was approximately 60%, irrespective of *TP53* aberrations (19). Although potentially curative, alloHSCT is only available for a highly selected young and fit CLL patient population, given the high risk of transplant-related morbidity and mortality. Thus, historically, treatment of patient with *TP53* aberrations has been challenging. Fortunately, alemtuzumab and alloHSCT have been replaced as treatment of choice for these patients by newer agents with greatly improved efficacy.

Advanced insight in the pathophysiology of CLL have led to the development of small molecule inhibitors that have revolutionized the treatment of patients with *TP53* aberrations. Ibrutinib is an irreversible inhibitor of Bruton’s tyrosine kinase (Btk), a signaling molecule downstream of the B cell receptor (BCR) (22–24). In the phase III RESONATE-2 trial, the efficacy of ibrutinib was compared with chlorambucil monotherapy (Clb) in unfit, treatment-naïve CLL patients (25–27). Although CLL patients with del(17p) were excluded from this trial, twelve ibrutinib-treated patients had mutated *TP53*. Their outcome following ibrutinib treatment was comparable to patients without *TP53* mutations (5-year PFS rate 56% *versus* 73%, HR 0.87; 95%CI, 0.26-2.85). Furthermore, in a phase II trial conducted by Farooqui et al., median PFS was not reached in *TP53* mutated CLL patients treated with ibrutinib monotherapy after a median follow-up of 35 months (28, 29). In the phase III ALLIANCE trial, previously untreated unfit CLL patients were treated with bendamustine rituximab (BR), ibrutinib alone or ibrutinib plus rituximab (R-ibrutinib). PFS was superior in all patients in both ibrutinib-based arms, compared with the BR arm, but this difference was most pronounced in patients with del(17p) (median PFS not reached [NR] after 38 months for both ibrutinib and R-ibrutinib *versus* 7 months for BR) (30). There was no difference in PFS for patients with del(17p) between the ibrutinib and R-ibrutinib arms (HR 1.57; 95%CI, 0.80-3.09). In the first-line phase III iLLUMINATE CLL trial, obinutuzumab plus ibrutinib (O-ibrutinib) was compared with obinutuzumab plus chlorambucil (O-Clb). In CLL patients with *TP53* aberrations, median PFS after O-ibrutinib was superior, compared with O-Clb (NR after 31 months *versus* 15.2 months, HR 0.14; 95%CI, 0.04-0.51) (31). Based on the evidence presented above, ibrutinib has thus become the gold standard for first-line therapy in patients with *TP53* aberrations. Interestingly, Brieghel et al. demonstrated that in ibrutinib-treated patients, the co-occurrence of multiple *TP53* aberrations (multi-hit) was associated with an inferior PFS and time-to-progression as compared with those with a single-hit (HR for PFS 14.1; 95%CI, 1.60-1849) (32). This observation has not yet been validated in larger cohorts, or in different treatment settings.

Importantly, ibrutinib monotherapy needs continuation until progression and is associated with adverse events such as fatigue, diarrhea, nausea, bleeding complications, cardiac arrhythmias and, rarely, sudden death (in 1% of treated patients) (33). In addition, in some population-based studies, adverse events were more severe resulting in a higher discontinuation rate, compared with those in clinical trials (34–36). The second-generation Btk-inhibitor acalabrutinib has a more selective binding profile and could therefore potentially overcome ibrutinib-associated toxicities. In the phase III ELEVATE-TN study, acalabrutinib and acalabrutinib plus obinutuzumab (O-acalabrutinib) were compared with O-Clb in unfit, treatment-naïve CLL patients (37). The estimated median 24-month PFS rate in patients with del(17p) was longer following treatment with O-acalabrutinib, compared with O-Clb (88%; 95%CI, 61–97% *versus* 22%; 95%CI, 5–45%). Similar results were obtained in patients with mutated *TP53* (24-month PFS rate 95%; 95%CI 70-99% *versus* 19%; 95%CI 5-41%).

Another small molecule inhibitor is the Bcl2-inhibitor venetoclax. Although this compound mainly acts through induction of apoptosis, it does so in a p53-independent manner (38). In the phase III GCLLSG CLL14 study, time-limited venetoclax plus obinutuzumab (Ven-O) was compared with O-Clb in previously untreated, unfit CLL patients. In patients with *TP53* aberrations, response and survival outcomes were better for Ven-O treated patients, compared with O-Clb treated patients (ORR 81% *versus* 36%, median PFS approximately 35 months *versus* 17 months, 24-month PFS rate approximately 96% versus 77%) (39–41). However, in the Ven-O arm, PFS for patients with *TP53* aberrancies was still inferior, compared with patients with intact *TP53* (HR 1.96; 95%CI, 0.92-4.17). Of note, none of the patients with *TP53* aberrations had progressive disease while receiving a therapeutic dose of venetoclax (39).

### R/R Setting

In the R/R setting, not surprisingly, chemoimmunotherapy yields disappointing results in patients with *TP53* aberrations. Badoux et al. reported poor response (ORR, 35%) and survival (median PFS 5 months and median OS 10.5 months) in R/R CLL patients with *TP53* aberrations after treatment with FCR (42). Consequently, R/R CLL patients with *TP53* aberrations require treatment with novel agent-based regimens.

In the R/R setting, Zelenetz et al. evaluated the efficacy of idelalisib (IDELA), a small molecule inhibitor of phosphoinositide 3-kinase (PI3K) in combination with BR (BR-IDELA) to treatment with BR alone. In patients with aberrant *TP53* signaling, ORR and PFS were superior after treatment with BR-IDELA, compared with BR alone (ORR: 58% vs 23%, median PFS 11.3 months *versus* 8.3 months, HR 0.47; 95%CI, 0.31-0.72) (43). However, when comparing patients with either del(17p) and/or *TP53* to patients with neither, median PFS after BR-IDELA remained poorer in patients with *TP53* aberrations (11.3 months *versus* 24.5 months) (43). Notably, in a R/R CLL cohort treated with rituximab and IDELA, followed by open-label, single-agent IDELA, the presence of *TP53* aberrations did not influence PFS (*TP53* aberrations: median PFS 20.8 months *versus* wildtype: 18.8 months, HR 1.03; 95%CI, 0.62-1.72) (44).

Several trials have demonstrated the impressive efficacy of ibrutinib in R/R CLL with *TP53* aberrations. In the phase III RESONATE trial, ibrutinib was compared with ofatumumab monotherapy in patients with R/R CLL (45–47). The 6-months PFS rate was 83% and 49% for patients with *TP53* aberrations treated with ibrutinib and ofatumumab, respectively (45). After six years of follow-up, ibrutinib-treated patients without *TP53* aberrations had a median PFS of 56.9 months (95%CI, 36.4-NR), compared to 40.7 months (95%CI, 25.4-57.3) for patients with either del(17p) or *TP53* mutations (48). Similarly, O’Brien et al. evaluated the efficacy of ibrutinib in R/R CLL patients with del(17p) in the RESONATE-17 trial (49). At 24 months, the ORR was 83% (95%CI, 76%-88%), with 63% of patients remaining progression-free (95%CI, 54%-70%). More recently, in the phase III ASCEND trial, acalabrutinib was compared with BR or R-IDELA in fit, R/R CLL patients (50). After a median follow-up period of 16.1 months, median PFS was NR and 16.5 months (95%CI, 14.0-17.1) for acalabrutinib and the investigators choice, respectively (HR 0.31; 95%CI, 0.20-0.49). Interestingly, very recently, the first head-to-head comparison of ibrutinib and acalabrutinib was performed (51). In this trial, Byrd et al. compared the efficacy of ibrutinib and acalabrutinib in R/R CLL patients. Acalabrutinib was non-inferior compared with ibrutinib in patients with del(17p): median PFS was 32.9 months (95%CI, 25.2-38.4) after treatment with acalabrutinib, compared with 27.6 months (95%CI, 21.8-28.5) for ibrutinib (HR 1.00; 95%CI, 0.73-1.38) (51).

Venetoclax has demonstrated comparable efficacy in R/R CLL with *TP53* aberrations. In the phase II Pivotal trial, venetoclax monotherapy was evaluated in R/R CLL patients with del(17p). The ORR was 77% for the overall cohort. The estimated 24-months PFS and OS were 66% (95%CI, 55%-74%) and 73% (95%CI, 65%-79%). In the phase III MURANO trial, venetoclax plus rituximab (Ven-R) was compared with BR in physically fit, R/R CLL patients (52–54). Outcome with Ven-R was superior in terms of median PFS as compared with BR in patients with del(17p) (NR after 48 months *versus* 15.4 months, respectively). However, in a pooled analysis of four early-stage trials, patients with either del(17p) or *TP53* mutations remained at higher hazard of relapse, following venetoclax-based treatment (HR 1.7; 95%CI, 1.2-2.4) (55).

Taken together, given the availability of more efficacious drugs, chemoimmunotherapy is no longer a suitable treatment option for CLL patients with *TP53* aberrations, both in first-line and in R/R settings. As a consequence, ideally every CLL patient with an indication for treatment should undergo cytogenetic and molecular testing for *TP53* disruption. At present, patients with *TP53* aberrations qualify for treatment with novel agents such as ibrutinib, acalabrutinib or venetoclax. Still, even after novel agent-based treatment, patients with *TP53* disruption seem to have inferior outcome, compared with patients with intact *TP53*, although these differences are not statistically significant in every trial. At present, there is no preference for either ibrutinib, acalabrutinib or venetoclax if considering treatment for patients with *TP53* aberrations. As such, the choice should be determined by the physician and the patient jointly, taking into account treatment duration, side effects, comorbidities and previous lines of therapy. A complete overview of clinical trials comparing treatment regimens in previously untreated or R/R CLL patients with *TP53* aberrations is given in **Table 1** and **Table 2**, respectively.

**Table 1:** An overview of clinical trials comparing first-line treatment regimens for patients with *TP53* aberrations.

Ref.	Trial	Authors	Year	Median FU	Treatment	Deletion 17p				No deletion 17p			
						N	ORR (95%CI)	mPFS (95%CI)	mOS (95%CI)	N	ORR (95%CI)	mPFS (95%CI)	mOS (95%CI)
(11, 12)	CLL4	Eichhorst et al.	2006	52.8	F or FC	28	–	23.3	29.2	261	–	62.2	84.6
(13)	E2997	Grever et al.	2007	–	F	9	–	8.9	–	20	–	14.1	–
					FC	10	–	11.9	–	17	–	NR	–
(14, 56, 57)	LRF CLL4	Catovsky et al.	2007	120	F, FC or Clb	55	27%	6	31.8	444	78%	26	73.8
(15, 16)	CLL8	Hallek et al.	2010	70	FC	29	34%	9.1	23	58	–	–	–
					FCR	22	68%	11.2	33.1	80	–	–	NR
(58)		Wierda et al.	2011	8	OFA-FC	8	83%	–	–	7	100%	–	–
(18)	CLL206	Pettitt et al.	2012	–	Alemtuzumab-MP	41	82%	11.8 (6.5-18)	23 (16.4-NR)	–	–	–	–
(59)		Strati et al.	2014	33	FCR-, R-, Len-based	63	63%	14 (10–18)	63 (43–83)	–	–	–	–
(25–27)	RESONATE-2	Burger et al.	2015	60	Clb	12	–	NR	–	112	–	NR	–
					OFA	3	–	–	–	91	–	–	–
(28, 29)		Farooqui et al.	2015	57	Ibrutinib	35	97% (86%-100%)	NR	NR	–	–	–	–
(17)		Rossi et al.	2015	70	FCR	30	–	22.5 (8.5-36.4)	–	48	–	58.9 (49.3-68.4)	–
(60, 61)	COMPLEMENT1	Hillman et al.	2015	28.9	Clb	39	–	3.7	18	343	–	12.9	NR
					OFA-Clb		–	12.8	NR		–	24	NR
(62)		O’Brien et al.	2015	22.4	R-IDELA	9	100%	NR	NR	52	96%	NR	NR
(63)		Le Bris et al.	2017	56.5	FCR	10	–	12	–	100	–	55	–
(64)		Mato et al.	2017	17	Ibrutinib	440	71%	36	–	181	–	NR	–
					IDELA	54	85%	12	–	8	–	16	–
(65)		Mato et al.	2018	17	Ven	56	71.4%	14		69	72%	NR	–
(66)		Takahashi et al.	2018	32.9	Len	9	–	6	34.6	89	–	55.9	98.2
(30)	ALLIANCE 041202	Woyach et al.	2018	38	BR	14	–	7	–	29	–	50	–
					Ibrutinib	9	–	NR	–	32	–	–	–
					R-Ibrutinib	11	–	NR	–	29	–	–	–
(39–41)	CLL14	Fischer et al.	2019	28.1	O-Clb	14	36%	15.1	NR	42	–	NR	NR
					O-Ven	17	81%	29	NR	50	–	NR	NR
(67)		Burger et al.	2019	36	Ibrutinib	41	80%	NR	–	35	74%	NR	
					R-Ibrutinib	36	97%	NR	–	49	86%	NR	
(31)	iLLUMINATE	Moreno et al.	2019	31.3	O-Ibrutinib	18	90%	NR	–	95	–	–	–
					O-Clb	23	68%	11.3	–	93	–	–	–
(68)	ELEVATE-TN	Sharman et al.	2020	28.3	O-Acalabrutinib	3	–	NR	–	11	–	NR	–
					Acalabrutinib	6	–	NR	–	20	–	NR	–
					O-Clb	16	–	13	–	77	–	23	–
(69)	GREEN	Stilgenbauer et al.	2021	43.7	O-mono	2	50%	15	NR	10	70%	35	NR
					O-Cbl	7	71.4%	20	30	10	80%	26	NR
					O-B	20	65%	22	45	61	86.9%	NR	NR
					O-FC	5	20%	10	30	20	100%	NR	NR

All follow-up is reported in months. 95%CI, 95% confidence interval; B, bendamustine; BR, bendamustine-rituximab; Clb, chlorambucil; F, fludarabine; FC, fludarabine; cyclophosphamide; FCR, fludarabine; cyclophosphamide and rituximab; FU, follow-up; IDELA, idelalisib; Len, lenalidomide; MP, methylprednisolone; mOS, median overall survival; mPFS, median progression-free survival; NR, not reached; OFA, ofatumumab; O, obinutuzumab; ORR, overall response rate; R, rituximab; Ven, venetoclax.

**Table 2:** An overview of clinical trials comparing treatment regimens for relapsed or refractory CLL patients with *TP53* aberrations.

Ref.	Trial	Authors	Year	Median FU	Treatment	Deletion 17p				No deletion 17p			
						N	ORR (95%CI)	mPFS (95%CI)	mOS (95%CI)	N	ORR (95%CI)	mPFS (95%CI)	mOS (95%CI)
(70)	CLLH	Stilgenbauer et al.	2009	37.9	Alemtuzumab	31	39%	5.8	18.3	18	50%	6.5	15.5
(42)		Badoux et al.	2011	43	FCR	20	35%	5	10.5	–	–	–	–
(45–47)	RESONATE	Byrd et al.	2014	74	Ibrutinib	63	90%	40.6 (25.4-44.6)	61.8 (38.7-NR)	132	91%	42.5 (31.7-56.2)	–
					OFA	64	12%	6	–	132	27%	9	–
(44, 71)		Furman et al.	2014	18	R-IDELA	46	–	18.7 (16.6-32.4)	–	64	–	20.8 (16.4-28.9)	–
					R	49	–	4.0 (3.7-5.7)	–	61	–	8.1 (5.1-8.2)	–
(72)	PCYC-1102	Byrd et al.	2015	32.5	Ibrutinib	34	79%	28.1	–	91	95%	NR	–
(73)		Thompson et al.	2015	28	Ibrutinib-based	43	94.1%	32	33	24	100%	NR	NR
(28)		Farooqui et al.	2015	57	Ibrutinib	16	80%	36	60	–	–	–	–
(74)		Jaglowski	2015	16.4	OFA-Ibrutinib	31	74.2% (55%-88%)	NR	–	39	87.2% (73%-96%)	–	–
(75)	CLL9	Bühler et al.	2016	24	Len	23	21.7%	4.9	18.9	70	47.1%	11	34.9
(76)		Roberts et al.	2016	17	Ven	31	71% (52%-86%)	16 (11–25)	–	60	80% (68%-89%)	25	–
(77)		Byrd et al.	2016	14.3	Acalabrutinib	18	100%	NR	–	43	–	–	–
(49)	RESONATE-17	O’Brien et al.	2016	27.6	Ibrutinib	144	83.3% (76.2-89.0)	NR (27.7-NR)	NR (29.5-NR)	–	–	–	–
(78)	M13-982	Stilgenbauer et al.	2016	12	Ven	107	74%	NR	NR	–	–	–	–
(43)		Zelenetz et al.	2017	14	BR + IDELA	69	58%	11.3	–	138	73%	24.6 (19.5-30.3)	–
					BR	68	23%	8.3	–	141	50%	11.2 (11.1-13.6)	–
(66)		Takahashi et al.	2018	32.9	Len	33	–	8.5	29	138	–	63.9	63.9
(79)		O’Brien et al.	2018	60	Ibrutinib	34	79%	26	57	92	–	NR	NR
(52–54)	MURANO	Seymour et al.	2018	48	Ven-R	46	–	NR	–	127	–	NR	–
					BR	46	–	15.4	–	123	–	21.4	–
(80)	Pivotal	Stilgenbauer et al.	2018	23.1	Ven	153	77%	27.2 (21.9-NR)	38.8	–	–	–	–
(81)		Bryd et al.	2020	41	Acalabrutinib	27	93% (76%-99%)	36 (21-NR)	–	–	–		
(50)	ASCEND	Ghia et al.	2020	16.1	Acalabrutinib	22	–	NR	–	132	–	NR	–
					BR or R-IDELA	13	–	13.8 (6.4-16.7)	–	141	–	16.9	–
(51)		Byrd et al.	2021	40.9	Acalabrutinib	121	–	32.9 (25.2-38.4)	–	147	–	–	–
					Ibrutinib	120	–	27.6(21.8-38.5)	–	145	–	–	–

All follow-up is reported in months. 95%CI, 95% confidence interval; BR, bendamustine-rituximab; FCR, fludarabine; cyclophosphamide and rituximab; FU, follow-up; IDELA, Idelalisib; Len, lenalidomide; mOS, median overall survival; mPFS, median progression-free survival; NR, not reached; OFA, ofatumumab; O, obinutuzumab; ORR, overall response rate; R, rituximab; Ven, venetoclax.

## Deletion of the Long Arm of Chromosome 11

Deletion of the long arm of chromosome 11, i.e. del(11q), is one of the most common structural chromosomal aberrations in CLL. At diagnosis, del(11q) is present in 10% of patients with early-stage and 25% of patients with advanced stage, treatment-naïve CLL (82, 83). Patients carrying a del(11q) characteristically have bulky disease, rapid progression and a shorter OS (82). The minimally deleted region in del(11q) encompasses several tumor suppressor genes such as *ATM, FDX, MLL* and *RDX*. The tumor suppressor gene *ATM* encodes the serine-threonine kinase ATM, which is important in the repair of double-strand DNA breaks. Deleterious *ATM* mutations in the residual allele can be found in 36% of the CLL patients with a del(11q) and are associated with a poorer prognosis, compared with patients with del(11q) alone (84–86). Since ATM functions as a positive upstream regulator of p53, loss of ATM could interfere with chemotherapy-induced apoptosis.

### First-Line Setting

Concordantly, in the chemotherapy era, prognosis for CLL patients with del(11q) was poor (83). In the US E2997 trial, first-line treatment with FC did not improve PFS in patients with del(11q), compared with F alone (median PFS 25.2 and 16 months, respectively). In the FC arm, PFS was 25.2 months and NR after a follow-up of 60 months for patients with del(11q) and those without, respectively. Moreover, in a multivariable analysis, del(11q) was strongly associated with reduced PFS (HR 1.904; *P*=0.006) (13, 87). Similarly, in the CLL8 trial, first-line treatment with FC resulted in a complete response (CR) rate of only 19% in del(11q) patients (15, 16). In contrast, in this trial, patients with del(11q) responded very well to treatment with FCR (CR rate 71%). In addition, the 5-year PFS rate for patients with del(11q) was 11.4% after FC, compared with 31.4% after FCR (HR 0.47; 95%CI, 0.32-0.68). Similarly, the 5-year OS rate of patients with del(11q) was superior after treatment with FCR, compared with FC (HR 0.35; 95%CI, 0.20-0.61). Indeed, subgroup analysis revealed that patients with del(11q) and mutated IGHV genes responded very well to FCR, with outcomes similar to patients without del(17p) and del(11q) (88). In a retrospective observational cohort study by Rossi et al., physically fit, treatment-naïve CLL patients were treated with FCR (17). After a median follow-up of 70 months, median PFS was 43.5 months (95%CI, 32.2-54.7) for patients with del(11q) and 56.9 months (95%CI, 47.1-66.6) for patients without del(11q) (*P*=0.01). In a multivariable analysis, del(11q) was identified as an independent predictor of PFS (HR 1.67; 95%CI, 1.13-2.46). Furthermore, in the GCLLSG CLL10 trial, a phase III non-inferiority trial comparing FCR to BR in physically fit, treatment-naïve CLL, patients with del(11q) had shorter PFS in the BR arm (HR 2.33; 95%CI, 1.47-3.67) (89). In summary, although the introduction of chemoimmunotherapy, especially FCR, has significantly improved the outcome of patients with del(11q), their prognosis after chemoimmunotherapy is still inferior, compared with patients without del(11q).

Treatment with ibrutinib has proven very effective in patients with del(11q), yielding outcomes similar to patients without del(11q) (25–27, 30, 90). For example, after first-line treatment with ibrutinib alone in the RESONATE-2 trial, the 60-month PFS rate was 79% in patients with del(11q), compared with 67% for patients without del(11q) (25–27). Surprisingly, pooled data from three phase III ibrutinib trials (RESONATE, RESONATE-2 and HELIOS) demonstrated that ibrutinib-treated patients with del(11q) had slightly longer PFS, compared with ibrutinib-treated patients without del(11q) (42-month PFS rate, 70% *versus* 65%, *P*=0.02) (91). In the trial by Shanafelt et al., PFS was superior for ibrutinib-R compared with FCR in treatment-naïve CLL patients with del(11q) (HR 0.24; 95%CI, 0.10-0.62) (90). Likewise, Woyach et al. demonstrated that in patients with del(11q) PFS was comparable after treatment with ibrutinib (median PFS NR) or ibrutinib-R (median PFS NR), but inferior after treatment with BR (median PFS 41 months; 95%CI, 36-NR) (30). In the ILLUMINATE trial, treatment-naïve CLL patients with del(11q) had significantly better PFS after treatment with ibrutinib (median PFS NR (95%CI, 17.4-NR), compared with O-Clb (median PFS 15.2 months (95%CI, 14.1-20.8). The ELEVATE-TN trial has demonstrated that after first-line treatment with acalabrutinib, PFS is comparable for patients with and without del(11q) (68). A complete overview of clinical trials comparing first-line treatment regimens for patients with del(11q) is provided in **Table 3**.

**Table 3:** An overview of clinical trials comparing first-line treatment regimens for patients with deletion 11q.

Ref.	Trial	Authors	Year	Median FU	Treatment	Deletion 11q				No deletion 11q			
						N	ORR (95%CI)	mPFS (95%CI)	mOS (95%CI)	N	ORR (95%CI)	mPFS (95%CI)	mOS (95%CI)
(13)	E2997	Grever et al.	2007	–	F	16	–	14.9	–	20	–	14.1	–
					FC	24	–	25.9	–	17	–	NR	–
(15, 16)	CLL8	Hallek et al.	2010	70	FC	69	87%	26	64	58	91%	–	–
					FCR	84	93%	50	NR	80	89%	–	–
(14, 56)	LRF CLL4	Catovsky et al.	2014	120	F, FC or Clb	71	–	16.8	57.6	374	–	–	–
(17)		Rossi et al.	2015	70	FCR	61	–	43.5 (32.2-54.7)	–	256	–	56.9 (47.1-66.6)	–
(25–27)	RESONATE-2	Burger et al.	2015	60	Ibrutinib	29	100%	NR	–	101	90%	NR	–
					Clb	25	–	9	–	108		18	–
(89)	CLL10	Eichhorst et al.	2016	37.1	FCR	68	99%	43.1	–	68	94%	–	–
					BR	63	90%	NR	–	76	97%	–	–
(30)	ALLIANCE 041202	Woyach et al.	2018	38	BR	33	–	41	–	134	–	50	–
					Ibrutinib	35	–	NR	–	137	–	NR	–
					R-Ibrutinib	37	–	NR	–	132	–	NR	–
(67)		Burger et al.	2019	36	Ibrutinib	27	89%	NR	NR	75	79%	NR	NR
					R-ibrutinib	15	87%	NR	NR	86	90%	NR	NR
(90)	ECOG-1912	Shanafelt et al.	2019	33.6	R-Ibrutinib	78	–	NR	–	69	–	NR	–
					FCR	39	–	NR	–	37	–	NR	–
(31)	iLLUMINATE	Moreno et al.	2019	31.3	O-ibrutinib	13	90%	NR	–	100	–	–	–
					O-Clb	22	68%	15.2	–	94	–	–	–
(39–41)	CLL14	Fischer et al.	2019	28.1	O-Clb	38	58%	18	–	42	80%	NR	
					O-Ven	36	81%	NR	–	50	84%	NR	
(68)	ELEVATE-TN	Sharman et al.	2020	28.3	O-Acalabrutinib	31	–	NR	–	148	–	NR	–
					Acalabrutinib	31	–	NR	–	148	–	NR	–
					R-Clb	33	–	17	–	144	–	28	–

All follow-up is reported in months. 95%CI, 95% confidence interval; BR, bendamustine-rituximab; Clb, chlorambucil; F, fludarabine; FC, fludarabine; cyclophosphamide; FCR, fludarabine; cyclophosphamide and rituximab; FU, follow-up; mOS, median overall survival; mPFS, median progression-free survival; NR, not reached; O, obinutuzumab; ORR, overall response rate; R, rituximab; Ven, venetoclax.

### R/R Setting

Treatment with chemoimmunotherapy in patients with R/R CLL with del(11q) is generally ineffective: in a study by Badoux et al. in the R/R setting, patients with del(11q) treated with FCR had a median PFS of only 12 months (42).Similar to the first-line setting, in R/R CLL with del(11q) treatment with novel agents has impressive efficacy. In the ASCEND trial, the 12-month PFS rate of patients with del(11q) after acalabrutinib was approximately 90%, compared with approximately 60% in patients with del(11q) after treatment with BR or R-IDELA (50). In the recent head-to-head trial in the R/R setting by Byrd et al., acalabrutinib was demonstrated to be non-inferior to ibrutinib in patients with del(11q) (51). In the CLL14 study, the presence of a del(11q) was associated with adverse PFS in the context of O-Clb (HR 3.44; 95%CI, 1.80-6.60), but not in the context of Ven-O (HR 0.94; 95%CI, 0.29-3.05) (39–41). Likewise, treatment with Ven-R in the MURANO trial resulted in a comparable PFS for R/R CLL patients with del(11q) (median PFS 48 months) and without del(11q) (median PFS 49 months) (52–54). A complete overview of all trials evaluating regimens for R/R CLL patients with del(11q) is provided in **Table 4**.

**Table 4:** An overview of clinical trials comparing treatment regimens for relapsed or refractory CLL patients with deletion 11q.

Ref.	Trial	Authors	Year	Median FU	Treatment	Deletion 11q				No deletion 11q			
						N	ORR (95%CI)	mPFS (95%CI)	mOS (95%CI)	N	ORR (95%CI)	mPFS (95%CI)	mOS (95%CI)
(70)	CLLH	Stilgenbauer et al.	2009	37.9	Alemtuzumab	20	30%	9	22.7	18	50%	6.5	15.5
(42)		Badoux et al.	2011	43	FCR	13	69%	12	33	–	–	–	–
(45–47)	RESONATE	Byrd et al.	2014	74	Ibrutinib	63	90%	60.7(36.4-NR)	–	132	90%	42.5 (31.7-56.2)	–
					OFA	59	12%	–	–	132	32%	–	–
(72)	PCYC-1102	Byrd et al.	2015	32.5	Ibrutinib	35	97%	38.7 (31.2-NR)	NR (41.2-NR)	66	85%	NR (NR-NR)	NR (NR-NR)
(76)		Roberts et al.	2016	17	Ven	28	82%	–	–	62	76%	–	–
(75)	CLL9	Bühler et al.	2011	24	Len	28	–	7.3	21.3	65	–	17.6	35.4
(92, 93)	HELIOS	Chanan-Khan et al.	2016	34.8	Ibrutinib-BR	23	–	NR	–	65	–	NR	–
					BR	55	–	11.73	–	172	–	16.36	–
(79)		O’Brien et al.	2018	60	Ibrutinib	36	–	51	NR	96	–	NR	NR
(52–54)	MURANO	Seymour et al.	2018	48	Ven-R	45	–	48	–	97	–	49	–
					BR	47	–	16	–	99	–	20	–
(50)	ASCEND	Ghia et al.	2020	16.1	Acalabrutinib	39	–	NR	–	116	–	NR	–
					BR or R-IDELA	44	–	17	–	110	–	28	–
(81)		Byrd et al.	2020	41	Acalabrutinib	21	95%	NR	–	107	95%	NR	–
(51)		Byrd et al.	2021	40.9	Acalabrutinib	167	–	38.4 (33.0-44.0)	–	101	–	–	–
					Ibrutinib	175	–	41.6 (38.0-44.8)	–	90	–	–	–

All follow-up is reported in months. 95%CI, 95% confidence interval; BR, bendamustine-rituximab; FCR, fludarabine; cyclophosphamide and rituximab; FU, follow-up; mOS, median overall survival; IDELA, Idelalisib; Len, lenalidomide; mPFS, median progression-free survival; NR, not reached; OFA, ofatumumab; O, obinutuzumab; ORR, overall response rate; R, rituximab; Ven, venetoclax.

In summary, in the historical context of chemotherapy, del(11q) was considered a marker of poor prognosis. Treatment with chemoimmunotherapy, specifically FCR, largely mitigates the deleterious outcome associated with del(11q), although survival of patients without del(11q) after FCR remains somewhat superior. Treatment with ibrutinib, acalabrutinib or venetoclax yields equal outcomes in patients with and without del(11q).

## Complex Karyotype/Genomic Complexity

The prognostic and predictive value of complex karyotype (CK) or genomic complexity (GC) in CLL has been reported in an increasing number of studies over the past decade. CK is traditionally defined as CLL with 3 or more cytogenetic aberrations, as measured by chromosomal banding analysis (CBA) or interphase FISH. When chromosomal aberrations are detected using chromosomal microarray analysis (CMA), the presence of 3 or more aberrations it is generally referred to as genomic complexity (GC). CBA and interphase FISH are complementary techniques (94). More specifically, CBA can detect abnormalities that cannot be found by a limited panel of FISH probes and FISH can detect abnormalities that cannot be found by CBA due to an inherently lower resolution (95–98). As such, performing CBA in combination with interphase FISH results in a more sensitive measurement of CK. Alternatively, CMA, often referred to as digital karyotyping, allows for screening the entire genome, and therefore has a higher detection rate of cytogenetic abnormalities, but may fail to detect cytogenetic aberrations without copy number aberrations, such as balanced translocations. Another advantage of CMA over CBA is that it is a DNA-based analysis and therefore culturing of B-cells in combination with mitogen stimulation is not needed to generate chromosome metaphases (99–101).

CK is associated with poor OS in CLL patients (98, 102–113). Indeed, in a large multicenter cohort study, Baliakas et al. confirmed that the presence of CK was associated with a shorter time-to-first-treatment (TTFT), compared with CLL patients with a normal karyotype (*P*=0.01) (114). Furthermore, TTFT was even shorter in patients harboring ≥5 chromosomal aberrations (*P*<0.001). The latter was confirmed in 2019, when the same research group proposed a hierarchical model for CK in which high CK (≥5 aberrations) exhibits the worst prognosis (median OS 3.1 years), compared with intermediate CK (4 aberrations, median OS 7.25 years) and low CK (3 chromosomal aberrations, median OS 12.3 years) (115). In that study, high CK emerged as a powerful prognostically adverse biomarker, independent of well-established prognostic indices. An exception to the former is posed by CLL patients that either have two translocations or trisomy 12 and trisomy 19, in combination with another cytogenetic lesion, which have a remarkably indolent disease course. The median survival of these patients was not reached after a median follow-up time of 7.1 years, and was superior compared with patients with no CK (median OS, 6.2 years, *P*<0.001) and patients with normal karyotype (median OS, 11.1, *P*<0.001). Although the impact of CK in CLL has been examined in many trials, there is no official consensus on the definition of CK. Consequently, it is challenging to evaluate the clinical significance of CK in the context of treatment. For example, several trials do not report on the minimum number of cytogenetic abnormalities required to define CK. Moreover, measurement techniques have not been standardized and it is often unclear whether only abnormalities that are present in a single clone are counted, or whether cytogenetic abnormalities in unrelated clones can also contribute to CK definition (116). Finally, patients with two translocations or trisomy 12 and 19 and another aberration, which have a more favorable prognosis, are rarely classified separately.

### First-Line Setting

Several trials indicate that CLL with CK (defined as ≥3 chromosomal aberrations) respond poorly to chemo-immunotherapy. In a study by Le Bris et al., in patients with previously untreated CLL treated with FCR, the presence of CK was associated with a significantly shorter median PFS (21 *versus* 55 months) and an inferior 5-year OS rate (72.4% vs 85.8%), compared with CLL patients without CK (63). In the R/R setting, Badoux et al. reported an ORR of 64% in patients with CK after treatment with FCR, with significantly shorter median PFS and OS compared with patients without CK (median PFS: 9 months *versus* 20.9 months, *P*<0.001, median OS: 26 months *versus* 46.7 months, *P*<0.001) (42). In a multivariable analysis, CK was associated with poorer PFS and OS independently of the presence of del(17p) (PFS: HR 2.6; 95%CI, 1.5-2.4 and OS: HR 1.9; 95%CI, 1.1-3.2). In the phase III GCLLSG CLL11 trial, the efficacy of Clb alone was compared with treatment with rituximab and chlorambucil (R-Clb) and O-Clb (117). In a subgroup analysis, Herling et al. reported on the impact of CK and coexisting mutations on the survival outcome of patients treated in this trial (118). Patients with CK pooled over all treatment arms, here defined as ≥3 aberrations by karyotyping, had inferior median OS, compared with patients without CK (37 months *versus* 60 months, *P*<0.001). This deleterious effect was largely dependent on patients with both CK and *TP53* aberrations, as their median OS was markedly poorer, compared with patients with CK and intact *TP53* (26 months *versus* 50 months). In a multivariable analysis, CK was a predictor for inferior OS, independent of well-established prognostic markers such as advanced clinical stage, unmutated IGHV and elevated β-2-microglobulin (HR 2.7; 95%CI, 1.4-5.3). The adverse impact of CK was retained when the model was limited to patients treated with chemoimmunotherapy (HR 2.6; 95%CI, 1.2-5.7) (118). In the CLL14 trial, first-line treatment with O-Clb resulted in significantly shorter PFS and OS in patients with CK, compared with those without (2-year PFS rate, 36.6% *versus* 69.6%, OS: HR 3.76; 95%CI, 1.36;10.29) (40). Contrastingly, in this trial PFS and OS were similar between patients with and without CK after treatment with Ven-O (2-year PFS rate: 78.9% *versus* 91.1%, HR. 1.91; 95%CI, 0.81-4.52 and 2-year OS rate: 88.2% and 93.2%; HR 1.51, 95%CI, 0.50-4.60) (40). Moreover, the presence of del(17p) and/or *TP53* mutation in patients with CK was associated with inferior PFS in the O-Clb arm (HR 2.10; 95%CI, 0.80-5.57), but not in the Ven-O arm (HR 1.42; 95%CI, 0.32-6.35).

### R/R Setting

Kreuzer et al. assessed the prognostic value of CK in R/R patients treated with R-IDELA (119). Interestingly, there was no impact of the presence of CK on ORR (80.8% *versus* 89.2%) and median PFS (20.9 *versus* 19.4 months; HR 1.22, 95%CI; 0.60-2.47). Similarly, Mato et al. found that R/R CLL patients with CK treated with IDELA had similar PFS to patients without CK (median PFS 9 months *versus* 12 months) (64).

Thompson et al. were the first to report on the impact of CK in the context of ibrutinib-based therapy (73). Although R/R CLL patients with and without CK had high ORR (90.5% *versus* 97.1%), median event-free survival (EFS) was significantly worse in patients with CK (19 *versus* 38 months; *P*<0.001). Importantly, in this trial, almost all patients with CK had an additional del(17p) (81%). Comparing patients with CK including or excluding del(17p) yielded a strong trend towards inferior median EFS in those with an additional del(17p) (22 months *versus* 34 months; *P*=0.056). OS was inferior in patients with CK, independently of the presence of del(17p) (HR 5.9; 95%CI,1.6-22.2). Likewise, in the RESONATE trial in R/R CLL, median PFS after treatment with ibrutinib was shorter in patients with CK, compared with those without (40.8 months *versus* 44.6 months, HR 1.292; 95%CI, 0.770-2.168) (45). Furthermore, with a median follow-up of 60 months, O’Brien et al. also demonstrated that R/R CLL patients with CK have inferior PFS and OS after treatment with ibrutinib, compared with patients without CK (median PFS: 31 months *versus* NR, median OS: 54 months *versus* NR) (49). In contrast, in a pooled analysis of three phase III ibrutinib trials for both treatment-naïve and previously treated CLL patients (the RESONATE-2, RESONATE and HELIOS trials), no effect of the presence of CK on PFS could be demonstrated (91). Specifically, the 42-month PFS rate was 63% in patients with CK, compared with 69% in those without (HR 1.02; *P*=0.95). Of note, the RESONATE-2 and HELIOS trials excluded patients with del(17p) (25, 92). Likewise, the ALLIANCE trial did not substantiate inferior survival in patients with CK after first line treatment with BR, ibrutinib and R-ibrutinib, compared with those without CK (HR 1.01; 95%CI, 0.68-1.51) (30). The impact of CK on treatment with acalabrutinib has only been evaluated in a single phase II trial in R/R CLL patients (81). PFS was shorter for patients with CK, compared with the overall cohort (33 months *versus* NR after 41 months).

The MURANO trial analyzed the impact of GC (≥3 cytogenetic aberrations, as measured by CMA) after treatment with Ven-R (53). In this trial, GC was stratified into low GC (3-4 aberrations) and high GC (≥5 aberrations). After treatment with Ven-R, patients without GC had superior PFS, compared with patients with low- and high GC (HR 2.9; 95%CI, 1.1-3.6). In addition, the co-occurrence of *TP53* aberrations only negatively affected PFS in patients with high CK, but not low CK. In concordance, in a pooled analysis of three phase I trials, Anderson et al. showed that the presence of CK was associated with shorter PFS in R/R CLL patients treated with venetoclax monotherapy (HR 6.61; 95%CI, 1.47-29.75) (120).

Altogether, the predictive impact of CK is challenging to disentangle, mostly due to inconsistent reporting and co-occurrence of *TP53* aberrations. While patients with CK seem to have a high chance of relapse, even in the absence of *TP53* aberrations, after treatment with chemoimmunotherapy, evidence of the impact of CK on the efficacy of novel agents such as ibrutinib and venetoclax is contradictory. Consequently, further research into the impact of CK is warranted, including clear reporting on the definition of CK and multivariable analysis to correct for the co-occurrence of *TP53* aberrations. For an overview of clinical trials comparing treatment regimens in CLL patients with and without CK, see **Table 5**.

**Table 5:** An overview of clinical trials comparing treatment regimens for patients with complex karyotype defined as ≥3 chromosomal aberrations.

Ref.	Trial	Authors	Year	Median FU	Treatment	Complex karyotype				Normal karyotype			
						N	ORR (95%CI)	mPFS (95%CI)	mOS (95%CI)	N	ORR (95%CI)	mPFS (95%CI)	mOS (95%CI)
(42)		Badoux et al.	2011	43	FCR	22	64%	9	26	–	–	–	–
(117, 118)	CLL11	Goede et al.	2014	40.9	Clb, R-Clb, O-Clb	30	–	–	37	124	–	–	60
(59)		Strati et al.	2014	33	FCR-, R- orLen-based	29	–	13	–	25	–	20	–
(45–48)	RESONATE	Byrd et al.	2014	74	Ibrutinib	39	90%	40.8 (22.5-44.6)	–	114	89%	44.6 (37.9-61.0)	–
					Ofatumumab	33	6%	–	–	114	33%	–	–
(73)		Thompson et al.	2015	28	Ibrutinib-based	21	90.5%	19	25	35	97.1	NR	NR
(63)		Le Bris et al.	2017	56.5	FCR	38	–	21	–	72	–	55	–
(120)		Anderson et al.	2017	23	Ven	11	–	16	–	19	–	NR	–
(64)		Mato et al.	2017	17	Ibrutinib	96	–	29	–	179		NR	
					IDELA	12	–	9	–	37		12	
(52, 53)	MURANO	Seymour et al.	2018	48	Ven-R	48	–	42	–	94	–	55	–
					BR	47	–	15	–	100	–	22	–
(65)		Mato et al.	2018	7	Ibrutinib	52	–	–	–	89	–	–	–
(79)		O’Brien et al.	2018	60	Ibrutinib	37	89%	31	54	64	–	NR	NR
(30)	ALLIANCE 041202	Woyach et al.	2018	38	BR	44	–	–	–	122	–	–	–
					Ibrutinib	39	–	–	–	126	–	–	–
					R-ibrutinib	60	–	–	–	108	–	–	–
(66)		Takahasi et al.	2018	32.9	Len-based	37	–	7.6	23	94	–	20.2	62.8
(119)		Kreuzer et al.	2019	29.2	R-IDELA	26	80.8% (60.6-93.4)	20.9 (8.5-NR)	28.3 (16.6-NR)	37	89.2% (74.6-97.0)	19.4 (16.4-28.9)	49.7 (25.5-NR)
					R-placebo	24	–	–	9.2 (2.0-53.5)	33	–	–	37.3 (16.0-NR)
(39–41)	CLL14	Fischer et al.	2019	28.1	O-Clb	30	50%	19.4		167	77.8%	NR	NR
					Ven-O	34	82.4%	NR		166	87.3%	NR	NR
(81)		Byrd et al.	2020	41	Acalabrutinib	20	90% (68%-99%)	33 (17-NR)	–	114	–	–	–

All follow-up is reported in months. 95%CI, 95% confidence interval; BR, bendamustine-rituximab; Clb, chlorambucil; fludarabine; cyclophosphamide and rituximab; IDELA, Idelalisib; FU, follow-up; mOS, median overall survival; Len, lenalidomide; mPFS, median progression-free survival; NR, not reached; O, Obinutuzumab; ORR, overall response rate; R, rituximab; Ven, venetoclax.

## Unmutated IGHV

The prognostic and predictive impact of the somatic hypermutation (SHM) imprint on the IGHV gene of the leukemia-specific BCR rearrangement has been recognized over the past two decades (121, 122). Whereas CLL with abundant SHM (IGHV germline identity <98%, ‘IGHV mutated’, or M-CLL) generally has an indolent disease course, a paucity of SHM (IGHV germline identity ≥98%, ‘IGHV unmutated’, or U-CLL) is a biomarker of high-risk disease and is associated with a shorter TTFT and OS, compared to M-CLL patients (121–123). Consequently, in early stage CLL, the proportion of patients with unmutated IGHV is around 50%, whereas this prevalence enriches to approximately 60% at first-line therapy and up to 80% in R/R CLL.

### First-Line Setting

After first-line treatment with FCR, a significant proportion of patients with M-CLL experience durable remission, whereas almost all patients with U-CLL eventually relapse (88, 124). For example, in the CLL8 trial U-CLL patients had significantly shorter PFS and OS following first-line treatment with FCR, compared with M-CLL patients (PFS: 5-year rate 33.1% *versus* 66.6%, OS: 5-year rate ~73% *versus* 83.6%, both *P*<0.001) (15, 16, 89). Similarly, in a trial by the MD Anderson Cancer Center the median PFS and OS of U-CLL patients after first-line FCR was 50.4 and 112.8 months, respectively, whereas median PFS and OS of M-CLL patients were NR after 12.8 years of follow-up (125). Additionally, the CLL10 trial demonstrated that the median PFS of U-CLL patients after treatment with FCR or BR was markedly shorter, compared with the median PFS of M-CLL patients (FCR: 42.7 months (95%CI,36.2-55.2) and BR: (95%CI, 33.6 months (30.3-38.4) in U-CLL, *versus* NR for both in M-CLL, *P*<0.001) (89).

Controversy remains as to whether chemoimmunotherapy or novel agent-based regimens are more appropriate as first-line treatment for patients with U-CLL. Head-to-head comparisons in U-CLL patients have demonstrated that, compared with chemoimmunotherapy, treatment with ibrutinib results in longer PFS. In the ALLIANCE trial, while U-CLL patients in both ibrutinib-based arms had similar median PFS (NR after 38 months), median PFS in the BR arm was markedly shorter (39 months, 95%CI,32-NR) (30). Likewise, the ECOG-1912 phase III trial compared the efficacy of R-ibrutinib to FCR in previously untreated CLL (90). In this trial, the 3-year PFS rate after FCR was 62.5% in patients with U-CLL, compared with 90.7% after R-ibrutinib (HR 0.26; 95%CI, 0.14-0.50). However, an OS benefit for patients with U-CLL after first-line treatment with ibrutinib-based regimens, compared with chemoimmunotherapy, has not been conclusively demonstrated. Consequently, guidelines differ on the most appropriate first-line regimen for U-CLL.

There is similar ambiguity regarding the most appropriate first-line treatment for unfit U-CLL patients, in which treatment with FCR or BR is contraindicated. In the CLL11 trial, first-line treatment with O-Clb resulted in significantly longer PFS, both compared with Clb alone (HR 0.23; 95%CI, 0.13-0.42) and R-Clb (HR 0.39; 95%CI, 0.29-0.53) in U-CLL (117). In the RESONATE-2 trial, the 18-month PFS of U-CLL patients was 89% after treatment with ibrutinib, compared with 47% after treatment with Clb (25). As both these trials demonstrated an OS benefit over Clb alone, treatment with O-Clb, R-Clb or ibrutinib has become the cornerstone of first-line therapy in unfit CLL patients. In a similar setting, U-CLL patients in the iLLUMINATE trial treated with O-ibrutinib had longer median PFS, compared with O-Clb (NR after 31.3 months *versus* 14.6 months, HR 0.15, 95%CI,0.08-0.27) (31). Newer agents have also been evaluated as first-line treatment for unfit CLL patients. In the CLL14 trial, treatment with Ven-O resulted in a superior 24-months PFS rate in U-CLL patients, compared with O-Clb (89.4% *versus* 51%, HR 0.22, 95%CI, 0.12-0.38) (40). Additionally, in the ELEVATE-TN trial, first-line treatment with O-acalabrutinib resulted in a 24-month PFS rate in U-CLL patients of 91% (95%CI, 83%-95%), compared with 31% (95%CI, 22%-40%) after O-Clb (68). However, none of these trials have so far demonstrated an OS benefit over O-Clb (51). Moreover, ibrutinib, venetoclax and acalabrutinib have not been compared in a head-to-head fashion in first-line setting. For these reasons, similarly to fit U-CLL patients, the most appropriate first-line treatment for unfit CLL patients remains controversial. In any case, all available options should be carefully discussed with the patient, taking into account the efficacy, contraindications, treatment duration and any side effects. For an overview of clinical trials comparing first-line treatment regimens in U-CLL, see **Table 6**.

**Table 6:** An overview of clinical trials comparing first-line treatment regimens for patients with U-CLL.

Ref.	Trial	Authors	Year	Median FU	Treatment	Unmutated IGHV				Mutated IGHV			
						n	ORR (95%CI)	mPFS (95%CI)	mOS (95%CI)	n	ORR (95%CI)	mPFS (95%CI)	mOS (95%CI)
(13)	E2997	Grever et al.	2007	–	F	53	–	15.0	–	60	–	23.4	–
					FC	57	–	31.4	–	65	–	NR	–
(41, 124)		Tam et al.	2008	153.6	FCR	126		50.4	112.8	88	–	NR	NR
(15, 16, 124, 126)	CLL8	Hallek et al.	2010	70.8	FC	194	76%	32.0	72.0	166	84%	41.9	NR
					FCR	196	91%	41.9	84.0		93%	NR	NR
(62)	101-08	O’Brien et al.	2015	36.4	IDELA	37	97.3%	NR	NR	37	95.7%	NR	NR
(72)	PCYC-1102	Byrd et al.	2015	35.2	Ibrutinib	15	87%	–	–	16	81%	–	–
(17)		Rossi et al.	2015	70	FCR	216	–	48.2 (43.7-52.7)	–	120	–	NR	–
(25–27)	RESONATE-2	Burger et al.	2015	60	Ibrutinib	42	95%	NR	NR	40	88%	NR	NR
					Clb	60	–	9	NR	42	–	17	NR
(89)	CLL10	Eichhorst et al.	2016	37.4	FCR	155	95%	42.7 (36.2-55.2)	–	196	95%	NR	–
				36.0	BR	108	95%	33.6 (30.3-38.4)	–	86	97%	55.4	–
(63)		Le Bris et al.	2017	56.5	FCR	77	–	36	NR	24	–	92	NR
(30)	ALLIANCE 041202	Woyach et al.	2018	38	BR	71	–	39 (32-NR)	NR	52	–	51 (51-NR)	NR
					Ibrutinib	77	–	NR	NR	45	–	NR	NR
					R-Ibrutinib	70	–	NR (48-NR)	NR	45	–	NR	NR
(90)	ECOG-1912	Shanafelt et al.	2019	33.6	R-Ibrutinib	210	–	NR	–	70	–	NR	–
					FCR	71	–	NR	–	44	–	NR	–
(31)	iLLUMINATE	Moreno et al.	2019	31.3	O-Ibrutinib	66	–	NR	–	41	–	–	–
					O-Clb	57	–	14.6 (11.1-15.1)	–	50	–	–	–
(39–41)	CLL14	Fisher et al.	2019	28.1	Ven-O	121	84%	NR	NR	76	85%	NR	NR
					O-Clb	123	63%	25.6	NR	83	85%	NR	NR
(68)	ELEVATE-TN	Sharman et al.	2020	28.3	O-Acalabrutinib	103	–	NR	–	76	–	NR	–
					Acalabrutinib	119	–	NR	–	60	–	NR	–
					O-Clb	116	–	20	–	61	–	NR	–
(69)	GREEN	Stilgenbauer et al.	2021	43.7	O-mono	28	71.4%	20	NR	23	69.6%	NR	NR
					O-Cbl	33	75.8%	26	NR	20	90%	34	NR
					O-B	180	82.2%	40	NR	107	71.3%	NR	NR
					O-FC	86	87.2	NR	NR	42	95.2%	NR	NR

All follow-up is reported in months. 95%CI, 95% confidence interval; B, bendamustine; BR, bendamustine-rituximab; Clb, chlorambucil; F, fludarabine; FC, fludarabine and cyclophosphamide; FCR, fludarabine; cyclophosphamide and rituximab; IDELA, Idelalisib; FU, follow-up; mOS, median overall survival; mPFS, median progression-free survival; NR, not reached; O, Obinutuzumab; ORR, overall response rate; R, rituximab; Ven, venetoclax.

### R/R Setting

In U-CLL patients with early relapse or refractory disease, chemoimmunotherapy usually yields disappointing results. In a trial conducted by Fischer et al., median PFS and OS in R/R U-CLL patients after treatment with BR were 13.8 and 25.6 months, respectively (127). Consequently, patients with R/R U-CLL usually require treatment with novel agent-based regimens. Two trials have demonstrated the efficacy of IDELA-based regimens in R/R U-CLL. In the trial operated by Furman et al., treatment with R-IDELA achieved a median PFS of 19.4 months in R/R U-CLL patients, compared with 5.6 months after rituximab alone (44, 71). In a similar setting, Zelenetz et al. demonstrated that R/R U-CLL patients have superior ORR, PFS and OS after treatment with BR-IDELA, compared with BR alone (ORR: 71% (95%CI, 63%-77%) *versus* 43% (95%CI, 35%-51%), median PFS: 19.5 months (95%CI, 16.1-24.6) *versus* 10.9 months (95%CI, 8.6-11.1), median OS: NR *versus* 31.6 months (95%CI, 22.2-NR) (43). However, due to the introduction of newer agents with better tolerability, IDELA is now used less frequently.

Several trials have demonstrated the impressive efficacy of ibrutinib in R/R U-CLL. In the RESONATE trial, the median PFS of U-CLL patients in the ibrutinib arm was 49.7 months (45–47). In the HELIOS trial, treatment with ibrutinib-BR for R/R U-CLL resulted in longer PFS, compared with BR (median PFS NR *versus* 13.8 months, HR 0.16; 95%CI, 0.11-0.21) (92, 93). Similarly, in the ASCEND trial, treatment with acalabrutinib alone achieved longer PFS in R/R U-CLL patients (median NR after 16 months), compared with BR (median 16.9 months; 95%CI, 11.6-NR) or R-IDELA (median 15.8 months; 95%CI, 13.9-17.1 months) (50). Recently, a head-to-head trial demonstrated that acalabrutinib and ibrutinib achieved similar PFS in R/R U-CLL patients (HR 1.09; 95%CI, 0.85-1.40) (51). Finally, venetoclax-based regimens are an efficacious option for R/R U-CLL. In the MURANO trial, R/R U-CLL patients achieved longer PFS after treatment with Ven-R, compared with BR (median PFS NR after 48 months *versus* 15.7 months, HR 0.16 (95%CI, 0.10-0.26)) (52). In R/R CLL, venetoclax, ibrutinib and acalabrutinib have not been compared in a head-to-head fashion.

In summary, for refractory or early relapsed U-CLL, novel agent-based regimens, either ibrutinib-, acalabrutinib- or venetoclax-based, are appropriate treatment options. For an overview of clinical trials comparing treatment regimens in R/R U-CLL, see **Table 7**.

**Table 7:** An overview of clinical trials comparing treatment regimens for patients with relapsed or refractory U-CLL.

Ref.	Trial	Authors	Year	Median FU	Treatment	Unmutated IGHV				Mutated IGHV			
						n	ORR (95%CI)	mPFS (95%CI)	mOS (95%CI)	n	ORR (95%CI)	mPFS (95%CI)	mOS (95%CI)
(70)	CLL2H	Stilgenbauer et al.	2009	37.9	Alemtuzumab	71	37%	8.0	18.6	22	32%	5.8	22.7
(42)		Badoux et al.	2011	43	FCR	59	96%	28	50	27	78%	44	NR
(15)		Fischer et al.	2011	24	BR	51	58.7%	13.8	25.6	25	78.2%	13.8	NR
(45–48)	RESONATE	Byrd et al.	2014	74	Ibrutinib	98	92%	49.7 (40.2-NR)	NR	36	89%	48.4 (35.6-60.8)	NR
					Ofatumumab	83	27%	–	NR	49	24%	–	NR
(44, 71)		Furman et al.	2014	18	R-IDELA	65	–	19.4	–	10	–	22.1	–
					R	75	–	5.6	–	13	–	8.5	–
(72, 79)	PCYC-1102	Byrd et al.	2015	61.5	Ibrutinib	79	91%	43	NR	16	81%	63	NR
(75)	CLL9	Bühler et al.	2016	24	Lenalidomide	69	39.7%	10.4	NR	20	45%	6.5	31.9
(76)		Roberts et al.	2016	17	Venetoclax	46	76%	–	–	17	94%	–	–
(92, 93)	HELIOS	Chanan-Khan et al.	2016	34.8	Ibrutinib-BR	67	–	NR	–	11	–	NR	–
					BR	178	–	13.8	–	28	–	24.6	–
(43)		Zelenetz et al.	2017	14	IDELA-BR	75	71% (63–77)	19.5 (16.1-24.6)	NR (26.8-NR)	9	68% (50–83)	26.4 (19.3-NR)	NR
					BR	127	43% (35–51)	10.9 (8.6-11.1)	31.6 (22.2-NR)	22	56% (38–72)	13.7 (8.3-18.5)	NR (15.2-NR)
(52–54)	MURANO	Seymour et al.	2018	48	Ven-R	123	–	NR	NR	53	–	NR	NR
					BR	123	–	15.7	NR	51	–	22.9	NR
(67)		Burger et al.	2019	36	Ibrutinib	61	92%	–	–	43	88%	–	–
					R-Ibrutinib	62	94%	–	–	42	95%	–	–
(50)	ASCEND	Ghia et al.	2020	16.1	Acalabrutinib	118	–	NR	–	33	–	NR	–
					BR or R-IDELA	125	–	16.2 (13.9-17.1)	–	26	–	18.3 (11.2-NR)	–
(81)		Byrd et al.	2020	41	Acalabrutinib	81	95% (88–99)	NR	–	30	–	NR	–

All follow-up is reported in months. 95%CI, 95% confidence interval; BR, bendamustine-rituximab; FCR, fludarabine; cyclophosphamide and rituximab; FU, follow-up; IDELA, Idelalisib; mOS, median overall survival; mPFS, median progression-free survival; NR, not reached; O, Obinutuzumab; ORR, overall response rate; R, rituximab; Ven, venetoclax.

## BCR Stereotyped Subsets

The molecular composition of the leukemia-specific BCR rearrangement has importance beyond its mutational status. Despite the immense theoretical variety of the BCR repertoire, subgroups of unrelated CLL patients express (quasi)identical leukemia-specific BCRs, a phenomenon known as BCR stereotypy. Although cumulatively, BCR stereotyped subsets are common, encompassing up to 41% of all CLL, the prevalence of each individual subset is low: the largest subset, subset #2 (defined as patients with a BCR comprised of IGHV3-21, IGLV3-21, with a short, stereotypic heavy-chain complementarity-determining region of 9 amino acids), represents around 2.5% of all patients (128). Certain stereotyped subsets have been associated with distinct clinico-biological profiles. For example, expression of a subset #2 stereotyped BCR is associated with poor prognosis, irrespective of IGHV mutational status, whereas patients from subset #8 have a very high risk of developing Richter’s syndrome (5-year risk: 68.7%) (129, 130).

Due to their low individual prevalence, little is known about the predictive impact of BCR stereotyped subsets. Jaramillo et al. analyzed the pooled results from the CLL8, CLL10 and CLL11 trials, which evaluated the efficacy of chemoimmunotherapy as first-line treatment for CLL (129). In these trials, compared with all other patients with mutated IGHV, patients with subset #2 and mutated IGHV had significantly shorter time to next treatment (TTNT) (HR 2.01; 95%CI, 1.23-3.28), numerically comparable to U-CLL patients. As of yet, there is no available data regarding the predictive impact of BCR stereotypy in the context of novel agent-based treatment regimens. Consequently, the therapeutic consequences of BCR stereotypy remain undefined, and testing for stereotypy has thus far not been embedded in regular CLL care.

## *NOTCH1* Mutated CLL

Activating mutations in *NOTCH1*, most often located in the PEST-domain or 3’-untranslated region (3’-UTR), are present in around 6-12% CLL patients at diagnosis and in 15-20% of patients with relapsed or refractory disease (131). CLL patients harboring a mutation in *NOTCH1* (*NOTCH1*-mut) have shorter OS, compared with their *NOTCH1*-wildtype (-wt) peers (132–134). Interestingly, some trials have provided evidence that *NOTCH1-*mut CLL patients may not benefit from treatment with an anti-CD20 mAb. In the CLL8 trial, *NOTCH1*-wt patients benefited significantly from inclusion of rituximab in their treatment regimen (FC: median PFS 32.8 months *versus* FCR: median PFS 57.3 months, *P*<0.001) *(* 16). Contrastingly, the median PFS of *NOTCH1*-mut patients did not improve significantly upon the addition of rituximab (FC: 33.9 months *versus* FCR: 34.2 months, *p*=0.9). Multivariable survival analysis yielded a statistically significant regression coefficient for the interaction term between *NOTCH1* status and treatment arm (HR 1.65; 95%CI, 1.076-2.535), thereby satisfying the formal criterion for a predictive variable (16). Concordantly, Dal Bo et al. demonstrated that *NOTCH1*-mut patients, in contrast to *NOTCH1-*wt patients, do not benefit from rituximab consolidation following treatment with fludarabine and rituximab (135). Finally, in the phase III COMPLEMENT1 study, which evaluated the efficacy of ofatumumab-Clb compared with Clb alone in unfit, treatment-naïve CLL patients, *NOTCH1*-mut patients did not benefit from the incorporation of ofatumumab in their treatment regimen (60). More specifically, the median PFS of *NOTCH-*wt patients treated with ofatumumab-Clb was longer, compared with those treated with Clb alone (23.8 months *versus* 13.3 months, HR 0.50; 95%CI, 0.39-0.63), whereas *NOTCH1*-mut patients had similar PFS, regardless of the treatment arm (17.2 months *versus* 13.1 months, HR 0.81; 95%CI,0.50-1.31). Again, a statistically significant interaction term between *NOTCH1* status and treatment arm provided evidence of the predictive impact (*P*=0.05) (60). Similar research for obinutuzumab, a type II anti-CD20 mAb, has not been performed and is warranted. PFS of *NOTCH1*-mut patients was similar to that of *NOTCH1*-wt patients in the RESONATE, CLL14 and MURANO trials, suggesting that *NOTCH1* status is not predictive in the context of novel agents (39, 46, 53). An overview of all clinical trials evaluating *NOTCH1* mutations in the context of therapy is given in **Table 8**.

**Table 8:** An overview of clinical trials comparing treatment regimens in patients with *NOTCH1* mutated CLL.

Ref.	Trial	Authors	Year	Median FU	Treatment	*NOTCH1* mutated				*NOTCH1* wildtype			
						n	ORR (95%CI)	mPFS (95%CI)	mOS (95%CI)	n	ORR (95%CI)	mPFS (95%CI)	mOS (95%CI)
(14, 56, 136)	LRF CLL4	Catosky et al.	2007	120	F, FC or Clb	46	–	22.0 (17.2-26.9)	54.8 (31.0-78.5)	420	–	26.4 (23.6-29.3)	74.6 (68.4-80.9)
(15, 16)	CLL8	Hallek et al.	2010	70.8	FC	62	87.1%	33.9	85.9	560	88.1%	32.8	83.7
					FCR		90.0%	34.2	79.2		96.6%	57.3	NR
(135)		Dal Bo et al.	2014	55	FR +/- R maint	20	90%	24	72	103	97%	88	126
(45, 46)	RESONATE	Byrd et al.	2014	74	Ibrutinib	43	–	NR	–	111	–	NR	–
(33, 90)	COMPLEMENT1	Hillmen et al.	2015	28.9	Clb	65	–	13.1	NR	318	–	13.3	NR
					Ofa-Clb		–	17	NR		–	23.8	NR
(63)		Le Bris et al.	2017	56.5	FCR	19		42	48	91		55	NR
(52, 53)	MURANO	Seymour et al.	2018	48	Ven-R	19	–	43		175	–	50	–
					BR	27	–	23	–	168	–	16	–
(39, 40)	CLL14	Fischer et al.	2019	28.1	Ven-O	47	78%	NR	–	164	–	NR	–
					O-Clb	48	62%	23.4	–	162	–	NR	–
(135)		Del Bo et al.	2020	25	Ibrutinib	65	–	26	38	115	–	NR	NR

All follow-up is reported in months. 95%CI, 95% confidence interval; BR, bendamustine-rituximab; Clb, chlorambucil; F, fludarabine; FC, fludarabine and cyclophosphamide; FR, fludarabine and rituximab; FCR, fludarabine; cyclophosphamide and rituximab; FU, follow-up; maint, maintenance; mOS, median overall survival; mPFS, median progression-free survival; NR, not reached; O, Obinutuzumab; Ofa, ofatumumab; ORR, overall response rate; R, rituximab; Ven, venetoclax.

## *BIRC3* Mutated CLL

Deleterious mutations in *BIRC3*, a negative regulator of non-canonical NF-κB signaling, are present in 3-5% of newly diagnosed CLL patients (84). Though relatively rare, *BIRC3* mutations are associated with poor outcome, with a 10-year OS rate in the chemoimmunotherapy era of just 29%, which was comparable to the OS of patients with *TP53* aberrations (137). Evidence from several trials suggests that patients with a *BIRC3* mutation (*BIRC3-*mut) respond poorly to chemoimmunotherapy. Firstly, *BIRC3* mutations are enriched in patients with fludarabine-refractory disease, with a prevalence of up to 24% (84). Furthermore, Diop et al. demonstrated that after treatment with FCR, *BIRC3*-mut patients had significantly shorter PFS, compared with patients without *BIRC3* mutations (*BIRC3*-wt) (median PFS 26.4 months *versus* ~54 months, *P*<0.001) (138). Moreover, in the CLL14 trial, the 24-month PFS rate of *BIRC3*-mut patients after treatment with O-Clb was considerably lower compared with *BIRC3-*wt patients, and similar to that of patients with del(17p) (*BIRC3-*mut: 14.3%, del(17p): 23.1%, all patients: 64.1%) (39, 40). Indeed, the ORR of *BIRC3-*mut patients after O-Clb was 38%, considerably lower than the ORR of the overall O-Clb arm (71%, *P*<0.05). In contrast, the presence of a *BIRC3* mutation does not seem to associate with inferior response to novel agents. In the Ven-O arm of the CLL14 trial, the 24-month PFS rate of *BIRC3*-mut patients was similar to the overall 24-month PFS rate in that arm (85.7% *versus* 88.2%). Similarly, in the RESONATE trial, PFS after treatment with ibrutinib for *BIRC3*-mut and *BIRC3*-wt patients was not significantly different (46).

Interestingly, *BIRC3* is located on chromosome 11q22, and is co-deleted together with *ATM* in 80% of CLL cases with del(11q) (85). In addition, genomic *BIRC3* defects mainly cluster in del(11q) patients, leading to biallelic loss of *BIRC3* (85). As such, the clinical significance of trials that report on the impact of mutated *BIRC3* need to be interpreted with caution, and ideally stratified for the presence of del(11q) to avoid confounding effects. Monoallelic loss of *BIRC3* did not influence survival after chemotherapy in the LRF CLL4 trial, whereas biallelic loss of *BIRC3* was associated with shorter OS (median OS 3.3 years *versus* 4.8 years, *P*=0.03) (56). Additional research on the predictive impact of monoallelic versus biallelic defects in *BIRC3* is warranted. An overview of all clinical trials evaluating *BIRC3* in the context of therapy is given in **Table 9**.

**Table 9:** An overview of clinical trials comparing treatment regimens in patients with *BIRC3* mutated CLL.

Ref.	Trial	Authors	Year	Median FU	Treatment	*BIRC3-*mutated				*BIRC3-*wildtype			
						n	ORR (95%CI)	mPFS (95%CI)	mOS (95%CI)	n	ORR (95%CI)	mPFS (95%CI)	mOS (95%CI)
(14, 56)	LRF CLL4	Catovsky et al.	2007	120	F, FC or Clb	28		20	72			24	72
(45–47)	RESONATE	Byrd et al.	2014	74	Ibrutinib	21	–	NR	–	133	–	NR	–
(39, 40)	CLL14	Fischer et al.	2019	28.1	Ven-O	7	82%	NR	–	204	–	NR	–
					O-Clb	9	38%	16.8	–	201	–	NR	–
(138)		Diop et al.	2020	81.6	FCR	9	–	26.4	–	278	–	54	–

All follow-up is reported in months. 95%CI, 95% confidence interval; Clb, chlorambucil; F, fludarabine; FC, fludarabine and cyclophosphamide; FCR, fludarabine; cyclophosphamide and rituximab; FU, follow-up; mOS, median overall survival; mPFS, median progression-free survival; NR, not reached; O, Obinutuzumab; ORR, overall response rate; Ven, venetoclax.

## New Pharmaceutical Perspectives for CLL Patients With High-Risk Molecular Features

Notwithstanding the significant advances in the field of CLL therapy, as detailed above, a proportion of CLL patients will exhaust all currently approved treatment options. In general, these are patients suffering from CLL with high-risk molecular features, such as aberrant *TP53* signaling, presence of CK/GC, or both. For these patients, a number of experimental treatments are currently under development (see **Table 10**).

**Table 10:** An overview of phase 1 and 2 trials of new drugs according to high-risk CLL subgroups.

Ref.	Authors	Year	Treatment	Setting	*TP53* aberrations			11q deletion			IGHV-unmutated		
					N	ORR (%)	CR (%)	N	ORR (%)	CR (%)	N	ORR (95%CI)	CR (%)
(139)	Sharman et al.	2017	Ublituximab + Ibrutinib	R/R	12	95	–	12	95	–	–	–	–
(140)	Tam et al.	2020	Zanubrutinib	TN and R/R	18	100		–	–	–	–	–	–
(141)	Tam et al.	2020	Zanubrutinib	TN with del(17p)	109	94.5	2.8	–	–	–	–	–	–
(142)	Xu et al.	2020	Zanubrutinib	R/R	22	86	–	20	82	–	51	82	–
(143)	Tam et al.	2020	Zanubrutinib + O	TN and R/R	16	88	–	10	–	–	19	–	–
(144, 145)	Davids et al.	2020	Duvelisib	TN and R/R	26	77	12	20	–	–	65	–	–
(146)	Davids et al.	2021	Duvelisib + FCR	TN	3	66	0	8	–	–	18	–	56
(147)	Mato et al.	2021	Pirtobrutinib	R/R	28	79	0	15	60	0	71	68	0
(139)	Sharman et al.	2021	Ublituximab + Ibrutinib	R/R	30	–	–	30	–	–	53	–	–

CR, complete response rate; del(17p), deletion of the short arm of chromosome 17; FCR, fludarabine; cyclophosphamide; rituximab; ORR, overall response rate; O, obinutuzumab; R/R, relapsed or refractory. TN, treatment naïve.

Zanubrutinib is a second-generation Btk inhibitor, which, compared with ibrutinib and acalabrutinib, has fewer off-target effects and a longer half-life. In a phase I trial in previously untreated or R/R CLL patients, zanubrutinib monotherapy resulted in an ORR in the overall cohort of 96.2%, and an ORR of 100% in patients with del(17p) (140). Moreover, in another phase I trial, the efficacy and safety of zanubrutinib plus obinutuzumab (O-zanubrutinib) was evaluated (143). This combination yielded an ORR of 100% in previously untreated CLL patients and 92% in R/R CLL patients. The ORR was 100% and 80% in treatment-naïve and R/R CLL patients with del(17p), respectively. The currently ongoing phase III SEQUOIA trial will evaluate the efficacy of zanubrutinib compared with BR in treatment-naïve CLL patients (148). Considering the poor outcome associated with any chemoimmunotherapy in patients with del(17p), those patients were not randomized in this trial but assigned to receive single-agent zanubrutinib, analyzed separately. Tam et al. published the primary report on safety and efficacy of the latter cohort (141). The ORR was 94.5%, with estimated 18-months PFS and OS of 88.6% (95%CI, 79%-94%) and 95.1% (95%CI, 88%-94%), respectively. The currently ongoing, phase III ALPINE trial will evaluate whether in R/R CLL, the efficacy of zanubrutinib is non-inferior, compared with ibrutinib (149).

To overcome treatment resistance against covalent Btk inhibitors, pirtobrutinib, previously known as LOXO-305, a non-covalent Btk inhibitor, has been developed. In the phase I/II BRUIN study, the safety and efficacy of pirtobrutinib were evaluated in R/R B-cell malignancies, including CLL (147). The ORR was 63% (95%CI, 55%-71%) for patients with CLL or SLL, irrespective of whether patients previously discontinued a covalent BTK inhibitor for progression, toxicity or other reasons. In patients with del(17p) and/or *TP53* mutation the ORR was 79%, compared with 60% in patients with del(11q) and 68% in patients with U-CLL. The currently ongoing phase III BRUIN CLL-321 trial will compare the efficacy of pirtobrutinib to either R-IDELA or BR in R/R-CLL patients that have been previously treated with a covalent Btk inhibitor (150).

Whereas IDELA only targets the delta subunit of PI3K, the novel agent duvelisib is a dual inhibitor of both the delta and gamma isoforms of PI3K. In the phase III DUO trial, duvelisib treatment was evaluated in patients who were progressive on ofatumumab (144, 145). Duvelisib treatment resulted in an ORR of 77%, with and median PFS of 15.7 months. Comparable responses were seen in patients with del(17p) and/or TP53 mutation (ORR, 77%, median PFS 14.7 months). Based on these results, the Food and Drug Administration (FDA) approved duvelisib for the treatment of R/R CLL and SLL in September 2018 (151). Duvelisib in combination with FCR was recently evaluated in young treatment-naïve CLL patients. The overall response was 88%. Hematological toxicity and infectious complications were common. Only three patients with *TP53* aberrations were identified, of whom two responded to the treatment (146).

Ublituximab is a next-generation, glyco-engineered, type I, anti-CD20 mAb that binds to a unique CD20 epitope which is different from the target site of rituximab, obinutuzumab and ofatumumab. In a phase II trial, ublituximab plus ibrutinib yielded an ORR of 88% in all R/R patients and 95% of patients with either *TP53* aberrations or del(11q) (139). In a recent, multicenter, phase III trial, ublituximab plus ibrutinib was compared with ibrutinib alone in R/R CLL patients (139). In the overall cohort, the ORR was 83% for ublituximab plus ibrutinib and 65% for ibrutinib alone (*P*=0.02). PFS was significantly longer in patients treated with ublituximab plus ibrutinib (HR 0.46; 95%CI, 0.24-0.87). In a subgroup analysis, PFS benefit was retained in patients with aberrant *TP53* signaling (HR 0.25; 95%CI, 0.10-0.65), but not in patients with del(11q) (HR 0.97; 95%CI, 0.36-2.61).

## Cellular Immunotherapy as New Perspective for CLL Patients With High-Risk Molecular Features

Although novel-based agents have revolutionized CLL therapy, they remain incapable of complete disease eradication. To this day, alloHSCT remains, in the context of CLL, the sole treatment with curative intent. Notwithstanding the availability of highly effective, highly tolerable agents, alloHSCT remains a relevant treatment option in several specified situations (152, 153). Currently, alloHSCT can be considered for patients with a relapse after chemoimmunotherapy, either in the presence of *TP53* aberrations (high-risk category 1) or with additional failure to BTK inhibitors and/or BCL2 inhibitors, irrespective of the presence of *TP53* aberrations (high-risk category 2) (153–155). In the high-risk category 1, the long-term benefits and risks of alloHSCT should be carefully balanced on an individualized basis. Specifically, a younger patient age (<65 years), absence of comorbidities and the availability of a suitable stem cell donor would argue in favor of an alloHSCT, whereas in the converse situation, novel-based agents would be more suitable. Contrastingly, alloHSCT is a more proportional treatment option for patients in high-risk category 2, considering their markedly poor prognosis, due to the limited availability of alternative options (154).

However, the field of immunotherapy has in recent years been revolutionized by the generation of chimeric antigen receptor (CAR) cytotoxic cells. These cells, most often T cells, are molecularly modified to express a single-chain antibody-variable fragment (scFab) with specificity for a marker that is ubiquitously expressed on the malignant target cells, fused to an intracellular CD3ζ domain. CAR efficacy has been further enhanced by the inclusion of a costimulatory domain, most often either CD28 or 4-1BB. CAR-T cells have been approved for the treatment of acute lymphoblastic leukemia, certain types of large B-cell lymphoma and multiple myeloma (156–158). Several investigators have evaluated the efficacy of CAR-T cells, most often directed against CD19, in the setting of R/R CLL (see **Table 11**). While the reported efficacy differs from study to study, the ORR, CR and median PFS reported in the larger studies (n≥10) are markedly lower (ORR: weighted mean 53%, range 38-71%, CR: weighted mean 26%, range 21%-29%, median PFS 3.1-7 months), compared with the impressive efficacy of CAR-T cell treatment in other lymphatic cancers (165, 169, 171, 174). One possible explanation for the unexpectedly low efficacy or CAR-T cell treatment in CLL is T cell exhaustion. In CLL patients, T cells express markers associated with T cell exhaustion, and the CAR-T cells generated from these source cells may have an impaired ability to kill malignant cells (177). Interestingly, ibrutinib has been found to boost T cell numbers and function in CLL, possibly through off-target effects on interleukin-2 inducible T cell kinase (ITK) or ZAP70, providing a rationale for concurrent treatment with CAR-T cells and ibrutinib (178). Indeed, Gauthier et al. treated 19 heavily-pretreated R/R CLL patients with anti-CD19 CAR-T cells and ibrutinib, achieving an ORR and CR of 83% and 22%, resulting in 1-year PFS and OS rate of 59% and 86%, respectively (172). An alternative approach to circumvent the problem posed by T cell exhaustion is using CAR-transduced natural killer (NK) cells. Allogeneic NK cells can be safely transfused irrespective of a full human leukocyte antigen (HLA) match, allowing the generation of an off-the-shelf, non-patient-specific CAR-construct generated from healthy donor cord blood. In a pilot study, Liu et al. treated 5 R/R CLL patients with anti-CD19 CAR-NK cells from HLA-mismatched donors (173). Of these five patients, three had a complete response, one had a partial response, and one patient did not respond and went on to receive a stem-cell transplantation. Although these results seem promising, both CAR-T cell therapy combined with ibrutinib treatment and CAR-NK cell therapy require more comprehensive evaluation in a larger cohort.

**Table 11:** An overview trials evaluating the efficacy CAR-T/NK cell trials in patients with CLL.

Ref.	Authors	Year	N	Setting	Ag Target	Costim. molecules	Lymphodepletion	Source	Concomitant drugs	CR (%)	ORR (%)	mPFS	mOS
(159)	Porter et al.	2011	1	R/R, TP53 ab	CD19	4-1BB	PC	Autologous	none	100%	100%	–	–
(160)	Brentjens et al.	2011	8	Chemorefractory	CD19	CD28	C (n=3)	Autologous	none	0%	0%	–	–
(161)	Kalos et al.	2011	3	R/R	CD19	4-1BB	PC or BR	Autologous	none	67%	100%	–	–
(162)	Kochenderfer et al.	2012	4	R/R	CD19	CD28	FC	Autologous	interleukin-2	25%	75%	–	–
(163)	Cruz et al.	2013	4	Relapse after HSCT	CD19	CD28	none	Allogeneic	none	0%	25%	–	–
(164)	Kochenderfer et al.	2015	5	R/R	CD19	CD28	FC	Autologous	none	60%	100%	–	–
(165)	Porter et al.	2015	15	R/R	CD19	4-1BB	B, FC or PC	Autologous	none	29%	57%	7	29
(166)	Fraietta et al.	2016	3	R/R	CD19	4-1BB	none	Autologous	Ibrutinib	33%	100%	–	–
(167)	Brudno et al.	2016	5	Relapse after HSCT	CD19	CD28	none	Allogeneic	none	20%	40%	3	–
(168)	Ramos et al.	2016	2	R/R	Igκ	CD28	BR or FR	Autologous	none	0%	0%	–	–
(169)	Turtle et al.	2017	24	R/R	CD19	4-1BB	F, C or FC	Autologous	none	21%	71%	–	–
(170)	Geyer et al.	2018	8	PR after first line CIT	CD19	CD28	C	Autologous	none	38%	25%	13.6	–
(171)	Geyer et al.	2019	16	R/R	CD19	CD28	B or C	Autologous	Ibrutinib (n=5)	25%	38%	3.1	17.1
(172)	Gauthier et al.	2020	19	R/R, Ibrutinib failure	CD19	4-1BB	FC	Autologous	Ibrutinib	21%	83%	–	–
(173)	Liu et al.	2020	5	R/R, CAR-NK cells	CD19	CD28	FC	Allogeneic	none	60%	80%	–	–
(174)	Frey et al.	2020	32	R/R	CD19	4-1BB	B, FC, PC, OFAO or GEMOX	Autologous	none	28%	44%	1	64
(175)	Shah et al.	2020	3	R/R	CD19+CD20	4-1BB	FC	Autologous	none	67%	100%	–	–
(176)	Cappell et al.	2020	7	R/R	CD19	CD28	FC	Autologous	none	63%	88%	40.5	–

All follow-up is reported in months. ab, aberrations; Ag, antigen; B, bendamustine; BR, bendamustine-rituximab; C, cyclophosphamide; CIT, chemoimmunotherapy; costim, costimulatory; CR, complete response rate; F; fludarabine; FC, fludarabine and cyclophosphamide; FR, fludarabine and rituximab; GEMOX, gemcitabine and oxaliplatin; HSCT, hematopoietic stem cell transplantation; mOS, median overall survival; mPFS, median progression-free survival; NK, natural killer; OFAO, oxaliplatin; fludarabine; cytarabine and ofatumumab; ORR, overall response rate; PC, pentostatin and cyclophosphamide; R/R, relapsed or refractory.

## Measuring Predictive Biomarkers in Clinical Care and Research: Author Recommendations

In this paragraph, based on the evidence presented above and summarized in the provided tables, we outline recommendations regarding when to measure the previously discussed predictive biomarkers. The powerful predictive impact of *TP53* aberrations is universally recognized and is presently incorporated in treatment decision algorithms in routine care. Consequently, *TP53* status should be both cytogenetically and molecularly assessed in all clinical trials and in all CLL patients with an indication for treatment. Although ambiguity remains whether the IGHV mutational status should influence the choice of first-line therapy, it can differentiate between patients with potential long-term remission and patients with a risk of earlier relapse after a time-limited highly effective first-line treatment regimen such as FCR. As such, we recommend assessment of the IGHV mutational status in all clinical trials and in all CLL patients with active disease. Accumulating evidence suggests that CK/GC could function as a predictive marker and is associated with poorer prognosis after chemoimmunotherapy. Although routine measurement of CK/GC may be desirable in clinical care, more evidence is required of the impact of CK in the context of novel agents before it can be incorporated in therapeutic decision-making. Consequently, we strongly recommend to perform CBA or CMA in all clinical trials, especially in trials evaluating novel agents. Additionally, measurement by CBA/CMA will detect the presence of more classical cytogenetic abnormalities, including del(17p) and del(11q), although the predictive relevance of the latter has significantly diminished since the introduction of chemoimmunotherapy and novel agents. The proposed resistance of *NOTCH1*-mutated CLL to treatment with rituximab and ofatumumab is intriguing and requires further research, especially focusing on the predictive impact of *NOTCH1* mutations in the context of regimens containing obininuzumab or ublituximab. Although the incorporation of *NOTCH1*-mutations in clinical care is not yet warranted, we recommend the assessment of *NOTCH1* mutational status in all trials evaluating regimens that include anti-CD20 mAbs. The predictive impact of BCR stereotypy and *BIRC3* mutations is currently unclear. As such, we do not recommend their routine assessment in clinical research, nor in patient care. As the prevalence of individual stereotyped subsets and *BIRC3* mutations is relatively low, the predictive impact of these biomarkers should be assessed either retrospectively in several pooled trials, or prospectively in specialized trials that specifically recruit patients with the molecular features of interest.

## Concluding Remarks

In this review, we have discussed treatment approaches to CLL with high-risk molecular features, providing a comprehensive overview of trials on this topic. Catalyzed by the advent of more advanced molecular techniques, our understanding of the pathophysiology of CLL has deepened over the years, leading to the identification of several cytogenetic, immunogenetic and molecular features that can differentiate between patients with low- and high-risk disease. Some of these, most notably *TP53* aberrations, have clear predictive impact and are presently incorporated in decision algorithms in routine care. Their presence is strongly associated with inferior response to chemoimmunotherapy and necessitates the use of novel agents. The predictive capability of other molecular features is less clear, especially in the context of novel-based treatment. The predictive importance of del(11q) has significantly diminished since the advent of chemoimmunotherapy and novel agents and is redundant in therapeutic decision-making. Despite accumulating evidence of a predictive impact from clinical trials, the lack of consistent reporting and standardization prohibits the current use of CK/GC in therapeutic decision-making. In fit patients, stratification by IGHV mutational status identifies patients who benefit markedly from treatment with first-line FCR, but whether U-CLL always warrants first-line treatment with novel agents remains controversial. Interestingly, some aberrations may be predictive in certain specific contexts only, such as the proposed resistance of *NOTCH1*-mutated CLL to treatment with rituximab and ofatumumab, but this observation requires further validation before *NOTCH1* status should be used to guide treatment choice. The data concerning the predictive impact of BCR stereotypy and *BIRC3* mutations are currently immature, and treatment choice should not dependent on the presence of these features. The place of second-generation novel agents and cellular immunotherapy in the treatment of CLL with high risk features is still elusive, but forthcoming data from early-stage trials is promising, necessitating further study. Of note, the vast majority of data concerning the predictive impact of the biomarkers discussed above has been obtained through prespecified or *post-hoc* subgroup analysis. While informative, these trials have not necessarily been powered to answer such questions, especially in the case of rare features. As such, there is an unmet need for randomized trials that evaluate the efficacy of treatments, especially of novel agent-based regimens, in cohorts of patients with pre-specified high-risk molecular features, to move further towards patient-tailored treatment strategies.

## Author Contributions

LvdS and PJH performed the collection and interpretations of all relevant literature. LvdS and PJH write the manuscript. APK, AWL and M-DL critically read and revised the manuscript. All authors contributed to the article and approved the submitted version.

## Conflict of Interest

The authors declare that the research was conducted in the absence of any commercial or financial relationships that could be construed as a potential conflict of interest.

## Publisher’s Note

All claims expressed in this article are solely those of the authors and do not necessarily represent those of their affiliated organizations, or those of the publisher, the editors and the reviewers. Any product that may be evaluated in this article, or claim that may be made by its manufacturer, is not guaranteed or endorsed by the publisher.
